# Evaluating the rancidity and quality of discarded oils in fast food restaurants

**DOI:** 10.1002/fsn3.1072

**Published:** 2019-06-06

**Authors:** Fatemeh Esfarjani, Khadijeh Khoshtinat, Aziz Zargaraan, Fatemeh Mohammadi‐Nasrabadi, Yeganeh Salmani, Zahra Saghafi, Hedayat Hosseini, Manochehr Bahmaei

**Affiliations:** ^1^ Food and Nutrition Policy and Planning Research Department, National Nutrition and Food Technology Research Institute, Faculty of Nutrition Sciences and Food Technology Shahid Beheshti University of Medical Sciences Tehran Iran; ^2^ Food Technology Department, National Nutrition & Food Technology Research Institute, Faculty of Nutrition Sciences and Food Technology Shahid Beheshti University of Medical Sciences Tehran Iran; ^3^ Department of Chemistry, Tehran North Branch Tehran Islamic Azad University (IAU) Tehran Iran

**Keywords:** discarded oils, fast food restaurants, quality assessment, rancidity

## Abstract

This cross‐sectional study attempts to determine the rancidity and quality of discarded oils in fast food restaurants. Samples of the discarded frying oils were collected randomly from 50 fast food restaurants in Tehran, Iran. Their physicochemical properties were assessed and compared to the standard values. The means (±*SD*) of the physicochemical indicators of the rancidity in the discarded oils were as follows: peroxide value, 3.06 (0.51) (mEq/kg); free fatty acids content, 1.52 (2.26) (%); *p*‐anisidine value, 57.63 (4.02) (mEq/kg); total oxidation value, 64.53 (4.15); total polar compounds (TPC), 20.19 (1.02) (%); viscosity, 107.87 (2.35) (cp); and red color, 9.64 (0.84). Positive correlations were found between the TPC, viscosity, and red color (*p* ≤ 0.01) of the oil samples. The majority of discarded oil from fast food restaurants were overdegraded containing hazardous secondary oxidative products, and also, the consumption of nonstandard frying oil has increased in fast food restaurants. Policymakers should develop guidelines to determine whether and when frying oils should be discarded and consider the consumption of overdegraded oils as a public health hazard.

## 1 INTRODUCTION

Frying is one of the most common methods used for preparation of foods throughout the world. Fast food is termed as a food obtained from restaurants and other catering establishments, where the aim is to provide a fast service and rapid customer turnover at reasonable prices. Fast foods include burgers, pizzas, sandwiches, and French fries (Sebastian, Ghazani, & Marangoni, 2014). They are easily served and prepared by energy‐efficient cooking methods and increase palatability due to fat absorption, crust formation, pleasant flavors, and odors. In Iran, like many developing countries, there has been a steady increase in the consumption of fried foods because of the high demand for such foods as a result of changing lifestyle, growth in the number of working women, and rapid expansion of fast food centers (Bahadoran, Mirmiran, Golzarand, Hosseini‐Esfahani, & Azizi, 2012; "Statistical Center of Iran, Household Expenditure and Income Statistics, 2016: www.amar.org.ir").

The physical and chemical properties of edible oil influence the degree of oxidation and hydrolysis reactions, which occur during frying. It is known that the frying oils used continuously or repeatedly at high temperatures in the presence of oxygen and water are subject to thermal oxidation, polymerization, and hydrolysis, and the resultant decomposition products can adversely affect the flavor and color of the foods. Their stability depends on the composition of fatty acids and natural antioxidants, as well as the frying temperature (Crosa et al., 2014).

During the frying process, different by‐products including alcohols, cyclic compounds, polymers, dimers, and free fatty acids are produced as a result of oxidation and hydrolysis reactions, which have adverse impacts on human health (Chen, Chiu, Cheng, Hsu, & Kuo, 2013). Also, they play important roles in the flavor, texture, and aroma acceptability of the fried products (Li, Ngadi, & Oluka, 2008). Due to the relatively high temperature of frying (150–180°C) and repeated heating, various undesired chemical reactions such as fission, hydrolysis, oxidation, polymerization, and pyrolysis occur very rapidly in triacylglycerol (Aladedunye, 2015; Zhang, Saleh, Chen, & Shen, 2012). These reactions result in other transformations in the physicochemical properties of the frying oil including its appearance (color, smell, foaming, viscosity, and density) and nutritional characteristics (increase in trans fatty acid [TFA] content, polar materials, and polymeric compounds and decrease in unsaturated fatty acid [USFA] compounds; Al‐Harbi & Al‐Kahtani, 1993). Consequently, various by‐products, such as esters, aldehydes, ketones, and peroxides that can be absorbed by the foods, are produced (Saguy & Dana, 2003). Peroxide is the first compound that is produced after oxidation of fats and oils. It can have negative impacts on human health and may contribute to different diseases such as cardiovascular diseases (CVDs), cancers, allergies, and obesity (Pizzino et al., 2017). During deep frying, different reactions occur depending on the factors such as replenishment of fresh oil, frying condition, and the original quality of the frying oil. Oil storage and cooking conditions can also produce a variety of materials due to oil oxidation and polymerization and thus contribute in the incidence of noncommunicable diseases (NCDs; Mozaffarian, Katan, Ascherio, Stampfer, & Willett, 2006; Oyagbemi, Azeez, & Saba, 2009). Also, some investigations about the negative effect of TFAs and SFAs on health, especially on coronary heart diseases (CHDs), have been conducted (Oomen et al., 2001).

Two irreversible processes including hydrolytic and oxidative rancidity determine the chemical stability of the frying oil, which affect the oil turnover during the frying process. Therefore, the quality of utilized frying oils and the factors that can influence their heat resistance are also important in order to monitor the quality of fried foods in fast food restaurants (Sebastian et al., 2014).

Various criteria are being used to judge when the frying oils need to be discarded. In restaurants and food services, changes in the physical properties of frying oils are considered as an indicator of oil quality. For instance, the frying oil may be discarded when it becomes dark, causes too much smoke, produces strong odor and greased texture, or when a persistent foam layer of the specified thickness is observed (Moreira, Castell‐Perez, & Barrufet, 1999). There is no agreement among different authors on the limits of color changes in frying oils because changes in their color depend on the oil variety, duration of exposure to light and heat, and the food itself. However, the information or protocol about discarded oils is limited.

The presence of metals in vegetable oils depends on several factors. They might come from the soil, environment, and genotype of the plant, fertilizers, and metal‐containing pesticides, introduced during the production process or by contamination from the metal processing equipment (Jamali et al., 2008).

To the best of our knowledge, the majority of studies conducted in Iran (Aalipour Hafshejani, Mahdavi, & Aalipour Hafshejani, 2015; Arbabi & Deris, 2011; Farrokhzadeh et al., 2013; Hassanzadazar, Ghayurdoost, Aminzare, Mottaghianpour, & Taami, 2018; Mohammadi, Hajeb, Seyyedian, Hossein Mohebbi, & Barmak, 2013; Navab Daneshmand & Ghavami, 2012; Pourmahmoudi, Akbartabar Turi, Poursamad, Sadat, & Karimi, 2008) have determined the frying oils’ rancidity by their peroxide value (PV). However, this study, for the first time, was conducted to do the analysis based on a set of physicochemical parameters (PV, free fatty acids [FFA], *p*‐anisidine value [*p*‐AV], total oxidation [TOTOX] value, red color, total polar compounds [TPC], and viscosity) and compared to the standard values reported by American Oil Chemists’ Society (AOCS), International Organization for Standardization (ISO), and Institute of Standards and Industrial Research of Iran (ISIRI) to evaluate the rancidity and quality of discarded frying oils in fast food restaurants and also to measure the amounts of their SFAs, TFAs, and heavy metal pollutants.

## 2 MATERIALS AND METHOD

### 2.1 Sample preparation

In this cross‐sectional study, samples of discarded frying oils were collected randomly from 50 fast food restaurants in five districts (north, east, west, south, and center) of Tehran, Iran. The districts were classified as high, moderate, and low socioeconomic status based on the Ministry of Economic and Financial Affairs’ report (Tajali‐pour & Alikhani, 2012). The oils’ status in fast food restaurants was assessed by conducting an interview with the fast food restaurants’ chefs about the used oil type, frequency of changing the oil in the fryer, frying methods, filtering methods, disposal mechanism of the discarded oil, the way of selling discarded oil, and the source of their knowledge about oil and frying procedure. The tests for quality control were based on the standards of AOCS, ISO, and ISIRI. According to the government standards of frying oil in Iran, the acceptable limit of PV, FFA, *p*‐AV, Ni, and Pb is 2 mEq/kg, 1%, 6 mEq/kg of oil, 0.5 and 0.1 ppm, respectively ((INSO), 2016b). The physicochemical properties of the fried oils including fatty acid profile, PV, FFA, *p*‐AV, TOTOX value, red color, TPC, and viscosity as the indicators of oils’ rancidity after frying were measured. The presence of heavy metals such as iron, lead, nickel, arsenic, and copper in the oils according to the ISIRI standards was also assessed ((INSO), 2016a; (INSO), 2018; AOCS.Cd8b‐90, 2011; [Link]18‐90; Masek, Latos, Chrzescijanska, & Zaborski, 2017).

The discarded oil samples were collected at the intervals of 10 days. To obtain homogeneous samples (900 ml) and reducing the errors, the samples were mixed by using a stainless‐steel spoon. Then, they were passed through the paper filter to a dark‐colored PET container (to prevent chemical changes). Subsequently, the encoded samples were transported in a cooler box with ice sheets to the food laboratory where the oil samples were filtered and stored at 4°C. The samples were analyzed after 16 hr. All measurements were replicated three times.

### 2.2 Determination of fatty acid profiles

Fatty acid methyl esters (FAMEs) were prepared from 0.5 g of oils according to ISO 12966 (2011). Then, they were analyzed using gas chromatography (GC) (Agilent 6890 GC, carrier gas Helium, flame gas H_2_, column HP‐88: 100 m) with a flame ionization detector (FID). The initial oven temperature started at 180°C, hold for 5 min, increased at the rate of 1°C/min to 190°C, hold for 20 min, then increased at the rate of 1°C/min to 200°C, and then hold for 17 min. The FID temperature was 220°C, the injection temperature was 210°C, and the retention time was 62 min. The quantification was done by comparing the peak area/height of lipid standards in the sample normalization system (“International Organization for Standardization (ISO), Animal, and vegetable fats and oils—Gas chromatography of fatty acid methyl esters,” 2011; Saghafi, Naeli, Tabibiazar, & Zargaraan, 2018).

### 2.3 Peroxide value (PV) measurement

The PV measurement was performed according to AOCS Cd 8b‐90 and expressed as mEq/kg of fat. For this purpose, 5 g of the oil sample was dissolved into 30 ml of acetic acid: isooctane (3:2) in a flask (AOCS.Cd8b‐90, 2011). The flask was swirled before the addition of saturated potassium iodide. The mixture was subjected to an excess of iodide via a saturated solution of potassium iodide (0.5 ml). The solution was swirled again for 1 min. The peroxides oxidized the iodide to iodine, and the iodine was titrated to a colorimetric endpoint (blue color disappeared) using 0.01 N sodium thiosulfate (Na_2_S_2_O_3_) solution (standardized using potassium dichromate and potassium iodide) with starch (10%) as an indicator. The amount of produced iodine was directly proportional to PV ((INSO), 2018).

### 2.4 Free fatty acids (FFA) content

Free fatty acids (FFA) are often used to assess the quality of frying oils (Chen et al., 2013). The high FFA values are due to triacylglycerol hydrolysis that takes place upon release of water from the food being fried. The determination of FFA is a simple titration to an endpoint of pH 8.3 with sodium hydroxide, and the results are expressed as %FFA (AOCS.Ca [Link]5a‐40).

### 2.5 *p*‐anisidine value (*p*‐AV) measurement

*p*‐Anisidine value (mEq/kg) was determined by a spectrophotometric method according to AOCS Cd 18‐90. One milligram of the oil sample was placed into a 25‐ml volumetric flask and diluted to volume with 5–10 ml isooctane. Then, the absorbance of the sample was measured at 350 nm as a blank using an ultraviolet/visible spectrophotometer (UV Mini 1240; Shimadzu Co.). Next, 5 ml of the solution was pipetted into the test tube and 5 ml of the isooctane solvent into the second test tube. One milliliter of the *p*‐anisidine solution was poured into each of the test tubes. After 10 min, the absorbance of the first test tube was measured at 350 nm using the second test tube as a reference. The *p*‐AV was measured by the formula given in the standard method (AOCS.Cd [Link]18‐90; Masek et al., 2017).

### 2.6 TOTOX value calculation

The total oxidation (TOTOX) value was calculated as TOTOX = 2PV + *p‐*AV, where PV and *p*‐AV represent peroxide value and *p*‐anisidine value, respectively (De Abreu, Losada, Maroto, & Cruz, 2010).

### 2.7 Color measurement

The color measurement was carried out using a Lovibond PFX 995 instrument, and the intensity of redness (*R*‐value) was measured according to AOCS Official Methods Cc 13b‐45. The oil samples were treated with colorful filter aid earth, agitated for 2.5 min, and then filtered ((INSO), 1998).

### 2.8 Total polar compounds (TPC) measurement

Total polar compounds was measured according to INSO (4087). The components of the oils and fats were determined by column chromatography under the conditions specified in the standard. To do this, the polar and nonpolar compositions of the test piece were separated by a column. Then, the nonpolar compounds were washed and weighed. As measuring the polar compounds according to INSO (4087) is time‐consuming and costly, the polar component metering device of the Tetso Company was used (Testo 270 instrument; Testo Company; Uriarte & Guillén, 2010).

### 2.9 Viscosity measurement

Viscosity as an indicator of polymerization after frying was measured using a DV‐I Prime viscometer, Spindle number 2 (Brookfield), and constant shear rate = 100 1/s (Kang & Yang, 2013; Liu et al., 2016).

### 2.10 Statistical analysis

After collecting the required data, the data analysis was performed by the SPSS software (version 16; SPSS Inc., Chicago, IL, USA). Data were summarized using frequency (%) for categorical variables, mean (±*SE*), and median (minimum–maximum) for normal and non‐normal distribution of data. Kolmogorov–Smirnov's test was used for checking the normality of the data. Between‐group comparisons were conducted using Kruskal–Wallis and Spearman's correlation matrix analyses. *p*‐Values <0.05 were considered as statistically significant.

## 3 RESULT

The results given in Table 1 show that most of the studied fast food restaurants (66%) add fresh oil to the old discarded oils during the frying process. Sixty‐eight percent of them use oil filtration in their kitchen. Metal filtration was the most used type of filter (36%). The majority of fast food restaurants replaced their discarded oil completely with fresh oil twice a week (54%), and the most important criterion for changing oil was color (84%). Previous cooking training course was the main source of information and awareness of the interviewees about fats and oils (48%).

**Table 1:** Frying oil practices by fast food restaurants^a^

Variables	Results
Adding fresh oil to the previous oil in the fryer	Yes: 66% (33) No: 34% (17)
Oil filtration (per day)	Once: 38% (19) Twice and more: 30% (15) Did not filter: 32% (16)
Filter type	Metal: 36% (18) Fabric and paper: 20% (10) Instrument: 12% (6) Did not filter: 32% (16)
Replacement with fresh oil	Daily: 8% (4) Three times a week: 28% (14) Twice a week: 54% (27) Once a week: 10% (5)
Most important criteria for changing the used oil	Color change: 84% (42) Decrease in volume: 10% (5) Burn odor: 6% (3)
Learning about oil and cooking	Training courses: 48% (24) Personal experiment: 34% (17) Seller of fryer: 18% (9)

a Brand names and the specifications of fast foods are reserved for the research team.

**Figure 1:**
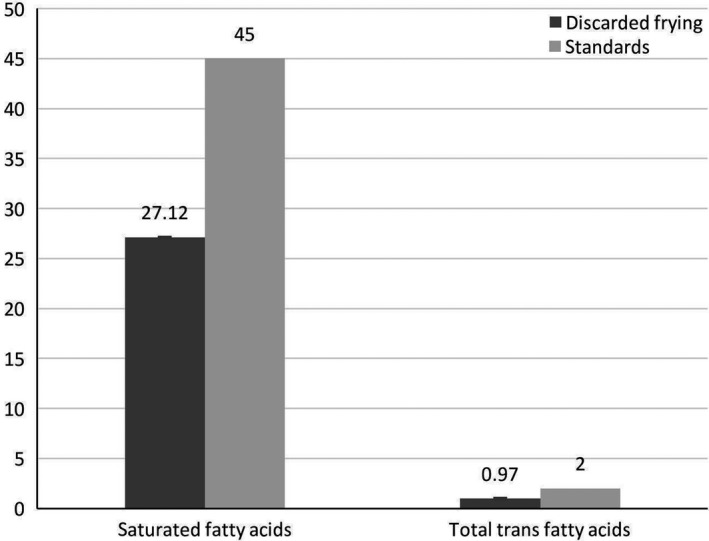
The mean of fatty acids in the discarded frying oil samples compared to the standards of the Iranian National Standardization Organization

**Figure 2:**
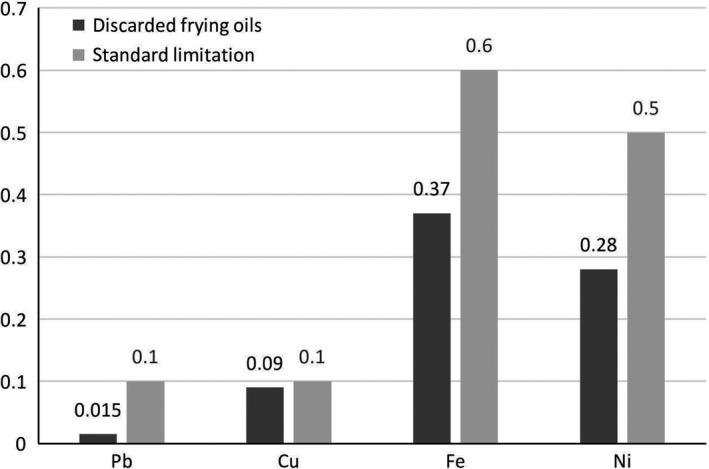
The mean of heavy metals in the discarded frying oils compared to the standards of the Iranian National Standardization Organization

Comparison of the fatty acid profile and heavy metals in the discarded oil samples of Tehran fast food restaurants with the standards of ISIRI is shown in Figures 1 and 2, respectively. In both comparisons, the fatty acid and metal contents were lower than the ISIRI standards. Table 2 indicates that the rancidity parameters of discarded oils were higher when they were changed once a week compared to daily one; however, they were not significantly different. The rancidity parameters in the majority of the fast food restaurants, which performed oil filtration, were lower than those that did not filter their oils (Table 3). The results of the Kruskal–Wallis analysis showed that the physicochemical parameters of the discarded oils were not significantly different based on the frequency of oil filtrations, too.

**Table 2:** Physicochemical analysis of discarded frying oils from fast food restaurants in Tehran based on oils’ fryers change

Variable	Daily (*n* = 4)	Three times a week (*n* = 14)	Twice a week (*n* = 27)	Once a week (*n* = 5)	Total (*n* = 50)
Peroxide value (mEq/kg)	2.05 (0.82)^a^	2.98 (0.97)	3.61 (0.87)	4.26 (2.50)	3.06 (0.51)
*p*‐AV (mEq/kg)	48.95 (12.74)	53.77 (5.47)	60.03 (7.59)	78.70 (12.69)	57.63 (4.02)
TOTOX (mEq/kg)	53.05 (12.34)	60.99 (5.77)	66.52 (7.65)	87.22 (11.99)	64.53 (4.15)
TPC (%)	18.70 (2.77)	21.67 (2.14)	19.50 (1.16)	21.50 (6.72)	20.19 (1.02)
Viscosity (cp)	102.64 (3.17)	110.61 (4.06)	107.43 (3.56)	107.80 (9.90)	107.87 (2.35)
Red color	8.94 (1.12)	10.95 (1.68)	10.15 (3.52)	9.34 (2.82)	9.64 (0.84)

Abbreviations: *p*‐AV, p‐anisidine value; TOTOX, total oxidation values; TPC, value for total polar compounds.

a Mean (*SE*).

**Table 3:** Physicochemical analysis of discarded frying oils from fast food restaurants in Tehran based on frequency of filtration

Variables*	Do filtration daily (*n* = 34)			No filtration (*n* = 16)
	Once (*n* = 19)	Twice or more (*n* = 15)	Total (*n* = 34)	
Peroxide value (mEq/kg)	3.44 (1.14)**	3.06 (0.64)	3.27 (0.69)	3.58 (1.15)
*p*‐AV (mEq/kg)	58.40 (6.67)	52.35 (7.25)	55.73 (4.87)	61.66 (7.28)
TOTOX (mEq/kg)	65. 29 (7.08)	58.96 (7.65)	62. 50 (5.15)	68.83 (7.07)
TPC (%)	18.52 (1.03)	19.26 (1.79)	18.85 (0.96)	23.03 (2.34)
Viscosity (cp)	104.06 (1.35)	110.98 (6.87)	107.11 (3.12)	109.48 (3.23)
Red color	0.93 (0.06)	0.88 (0.07)	9.42 (1.06)	10.09 (1.40)

Abbreviations: *p*‐AV, *p*‐anisidine value; TOTOX, total oxidation values; TPC, total polar compounds.

* Kruskal–Wallis analysis did not indicate the significant difference.

** Mean (*SE*).

**Figure 3:**
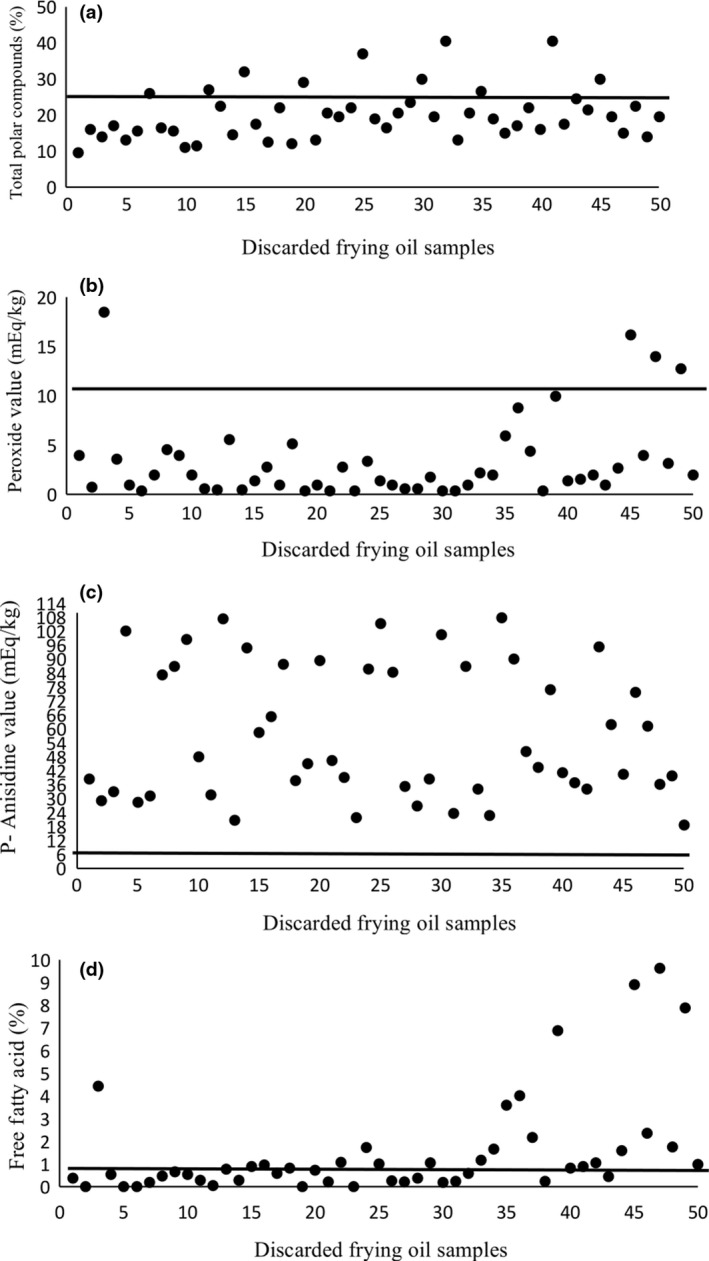
Rancidity measurements of the discarded frying oil samples from fast food restaurants. (a) Total polar compound; (b) peroxide value; (c) *p*‐anisidine value; and (d) free fatty acid

Figure 3 illustrates the chemical measurements of rancidity in the discarded frying oil samples obtained from fast food restaurants. The majority of discarded frying oil samples had acceptable TPC value and FFA based on INSO limits. The average FFA levels for discarded oil samples showed wide variations in FFA levels, from 0.01% to 9.64%. Since there is not any standard (limited line) for PV and *p*‐AV for discarded oils in Iran, the limit line of these parameters is based on usability limits of edible oil.

A correlation analysis of the given parameters is shown in Table 4. There was a correlation (*p* < 0.01) between TPC and viscosity and between TPC and red color. The PV values were also correlated to TOTOX and red color. Furthermore, there was a significant correlation (*p* < 0.01) between TOTOX and *p*‐AV.

**Table 4:** Correlation analysis of the physicochemical parameters of discarded frying oils from fast food restaurants in Tehran

Variables*	Peroxide value	*p*‐AV	TOTOX	TPC	Viscosity	Red color
Peroxide value (mEq/kg)	1					
*p*‐AV (mEq/kg)	0.144	1				
TOTOX (mEq/kg)	0.306*	0.958**	1			
TPC (%)	0.077	0.245	0.242	1		
Viscosity (cp)	−0.149	0.254	0.232	0.602**	1	
Red color	−0.524**	−0.105	−0.157	0.387**	0.192	1

Note

Values represent the Spearman's correlation coefficient (*r*) for the linear analysis.

Abbreviations:* p‐*AV, *p*‐anisidine value; TOTOX, total oxidation values; TPC, total polar compounds.

* Correlation is significant at the 0.05 level (2‐tailed).

** Correlation is significant at the 0.01 level (2‐tailed).

## 4 DISCUSSION

Different factors including price, acceptable shelf life, acceptable organoleptic properties, and nutritional value can affect the oil selection. Storage conditions including light, temperature, and oxygen pressure can have impacts on the shelf life of frying oils (Vitrac, Trystram, & Raoult‐Wack, 2000).

The qualitative findings indicated that most of the fast food restaurants used to replenish the fryers with fresh oil during the frying and did oil filtration once a day. The majority of fast food restaurants replaced their discarded oil completely with fresh oil twice a week due to its color change. In fast food restaurants, the oil's quality is usually monitored based on its color and odor, as well as the taste of fried food (Sebastian et al., 2014). A study in the United Kingdom showed that a certain way to determine that oil has degraded to the point of discard is when food begins to have an “off” odor or taste. This may manifest itself as a bitter taste, especially a bitter after‐taste (McSavage & Trevisan, 2001). The main source of information about the interviewees about fats and oils was the cooking course and training. Overall, it seems that the cooking course does not provide adequate information about desirable cooking practice and food safety.

The mean PV of the discarded oils examined in the present work was 3.06, which is higher than the sufficiency to use oils recommended by INSO (≤2). This can be caused by many factors such as oil type, amount of antioxidants, storage life and conditions, and also temperature in the frying process. Several similar studies in Iran have also shown that the PV index for discarded oils was higher than the standard limit (Arbabi & Deris, 2011; Farrokhzadeh et al., 2013; Mohammadi et al., 2013; Pourmahmoudi et al., 2008). The results of a study in Iran (2018) showed that more than 80% of oil samples had higher range of PV and AV than the recommended limits of INSO (Hassanzadazar et al., 2018). PV gives the initial evidence of rancidity in unsaturated fats. It is also a measure of the extent to which an oil sample has undergone primary oxidation, especially during storage. Freshly refined oils usually have a PV lower than 1 mEq/kg oil, while at a PV of above 10 mEq/kg oil, the oil is considered to be oxidized (Gunstone, 2008; Sebastian et al., 2014). It has been shown that an increase in temperature can cause a reduction in PV due to the decomposition of hydroperoxides. The amount of production and failure of hydroperoxides in oil depends on the temperature, time, and composition of the fatty acids. Because peroxide test is a function of the unstable nature of hydroperoxides, it cannot be used solely as an accurate indicator of the amount of oxidation of oils and fats (Navab Daneshmand & Ghavami, 2012). In fact, the primary oxidation products rapidly break down into secondary oxidation products, and thus, their total accumulation in the oil could be greatly underestimated (Kubow, 1992). Therefore, PV may not indicate the actual extent of oil deterioration and is not recommended for measuring oil deterioration during the frying process (Farhoosh & Moosavi, 2010).

The Iranian National Standardization Organization requires that fryer oil should be discarded when FFA levels in the oil exceed 1% ((INSO), 2016b). More than 36% of our discarded oil samples had FFA levels above 1%. The United States Department of Agriculture and some European countries have regulatory guidelines on maximum FFA levels in frying oil, ranging from 1.0% to 2.5%, depending on the regulations of a given country (Bailey & Shahidi, 2005; Sebastian et al., 2014). In this study, the maximum level of FFA content for discarded oil was 9.64%.

According to the standard of INSO, a desirable *p*‐AV for fresh frying oil is <4.0, with an upper limit of 6.0 ((INSO), 2016b). At a *p*‐AV above 6, the oil would be highly oxidized. According to our findings, *p*‐AV of the discarded samples was 58 that can result in higher oxidation. Determination of *p*‐AV is considered to be the most accurate and reliable method to assess the oxidative state of oils (van der Merwe, du Plessis, & Taylor, 2004; Sebastian et al., 2014). This measurement requires laboratory procedures and a number of simple instrumental methods suitable for use in a food processing factory. Combination of *p*‐AV with PV (TOTOX = 2PV + *p‐*AV) can indicate the overall oxidation state of oils over time. In general, the recommended standard TOTOX value is less than or equal to 19.5 mEq/kg, which increases linearly with both PV and *p*‐AV (De Abreu et al., 2010). Similar to the findings of De Abreu et al., the findings also showed that the TOTOX value in the discarded oil samples had an increasing trend (range 23–120).

The present research results revealed that the mean (±*SD*) of TPC was 20 (±7)%, which is acceptable according to INSO recommendations ((INSO), 2016b). Several reports and standards have reported the maximum level of TPC for oils to be discarded as 25%–27% (Gertz & Stier, 2011; Uriarte & Guillén, 2010). However, more researches are needed to find the threshold level at which frying oils should be discarded (Melton, Jafar, Sykes, & Trigiano, 1994). In Europe and many other places of the world, TPC is used as a regulatory index for determining when frying oil should be discarded (Sebastian et al., 2014). It seems that TPC test does not provide an accurate estimate of the oils’ deterioration; therefore, other reliable factors such as *p*‐AV should be considered too.

During the frying process, some polymerization of the fat may occur. In some cases, this leads to foam formation. Polymerization (thermal or oxidative) can affect the greasiness of fried foods. The polymerization values of 0.5%–5.36% have been reported for commercial samples of used frying oils (Orthoefer & List, 2007). In the present study, the mean of discarded oil viscosity was increased (108 cp), which is an undesirable characteristic. Increasing the viscosity is an indicator of the level of polymerization in high oxidation stage of oils (Kang & Yang, 2013).

The findings of this study indicated that TFAs, UFAs, and heavy metal pollutants were in the acceptable range according to the INSO standards. Since oil is subject to mandatory pricing in Iran and the commercial price is relatively high and not cost benefit for the producers, its production has TFAs, as indicators of hydrogenated oils, in most of the discarded oil samples, may show that the usage of nonstandard frying oils for guilds and industrial applications. The evaluation of fatty acid profiles in the studied discarded frying oils showed that in the formulation of fresh frying oils used in fast food restaurants, a larger volume of soybean oil than palm oil is used. Since soybean oil has very low resistance due to having high amount of UFAs with dual bands and thus reduces the oxidative stability of the oil, it is not suitable for frying; also because of producing aldehydes, the probability of heart and cancer diseases increases. Although palm oil has higher SFAs than other types of vegetable oils, it has a significant role in improving the physicochemical properties, oxidative stability, and quality of frying oils (Amirsardari, Asadollahi, & Eshaghi, 2018; Shahidi, 2005). It seems that, because of easy access and low price, the usage of soybean oils in fast food restaurants has increased.

In general, concerning the policies of Iran's Ministry of Health and Medical Education (MOHME), the content of total TFA of edible oils and fats has considerably reduced from 18.2% in 2006 to 1.36% in 2013. Furthermore, the plans of MOHME were very effective in reducing TFA intake via edible oils in the Iranian population. Regarding the World Health Organization (WHO) recommendation to reduce the risk factors of NCDs, and due to increase in the incidence of CHD in Iran, it is necessary to design a comprehensive study for determining the total dietary intake of TFA from all risky sources among the Iranian population, as well as identifying its possible effects on human health (Abedi et al., 2016).

A comparison between the given parameters showed the correlation of TPC value with viscosity and red color. The viscosity of an oil is dependent upon its TPC value and so are its electrical properties; therefore, it seems plausible that the viscosity of oil can be estimated from its electrical properties. The advantage of knowing such a relation would enable to determine the viscosity of oil by measuring only its electrical properties (Kumar, Singh, & Tarsikka, 2013). Also, there was a weak correlation between *p*‐AV and the other parameters because *p*‐AV targets secondary oxidation products, while the rest of the parameters are more sensitive to both the primary oxidation and the hydrolysis stages of frying oil breakdown (Karimi, Wawire, & Mathooko, 2017; Sebastian et al., 2014).

This study indicated that the majority of discarded oil from fast food restaurants in Tehran were overdegraded containing hazardous secondary oxidative products. Due to the potential toxicity of oxidation products, this could pose a public health hazard (McSavage & Trevisan, 2001). Frying oil should be considered as a food item because fried foods will absorb a significant amount of oil, and there are many hazardous degradation products present in them. The high values of degradation products found in frying oils raise concerns about the safety of the foods served in some fast food restaurants in Tehran and anywhere in the world. In this context, it would seem appropriate to suggest to local public health authorities include frying oil safety monitoring as part of the food premises’ health inspection process. The 4th International Symposium on Deep‐Fat Frying has recommended the implementation of such regulatory guidelines to protect public health (Boons & Mendoza, 2010). Unfortunately, there are no such guidelines or regulatory standards for frying oils in most of countries. There are several methods available for analyzing the heat abuse of oils. Usually, cooks decide on the quality of frying oils by visual observations such as color, excessive foaming, and smoking. Another assessment is based on odor and taste of the fried foods, as well as the cost of buying fresh oil. Therefore, it is essential to monitor the quality of oils to avoid the health consequences of consuming foods fried in degraded oils. Hence, it is highly recommended to inspect the performance of oil quality in fast food restaurants and food industry establishments and create regulatory standards for the quality of frying oils (Zargaraan et al., 2018).

The new rules of Food Safety and Standards Authority of India (FSSAI) are now prohibiting cooking more than three times using the same oil. To ensure that the rule is followed, eateries will be required to keep a log of their purchase and use of edible oils since 2017. The FSSAI has directed all Food and Drug Administrations (FDA) across the country to notify restaurants using more than 50 L of oil per day to maintain a daily chart. As per the new rules, the TPC limit is 25%, beyond which the oil is not suitable for use. A regulation has been set by FSSAI to ensure such used cooking oil is neither directly used in the food preparation nor re‐entered into the food chain. Per this regulation, all Food Business Operators whose consumption of edible oils for frying is more than 50 L per day shall maintain the records and dispose used cooking oil to agencies authorized by the FSSAI or Commissioner of Food Safety of States/UTs from time to time (FSSAI, 2017). This is a simple lesson from India that should be considered in fast food restaurants.

Further research is needed to find such rapid multiparameter test kits in order to establish the quality of reused oil and the point at which it should be discarded in fast food restaurants and also more appropriate methods to monitor the quality of frying oils (Bansal et al., 2010).

So it needs to develop rapid, simple, convenient‐to‐use, and powerful tests for checking deterioration of the oil. Using this method, it would be possible to monitor oil quality online; yet, it is necessary to develop policies and guidelines that can be used to determine whether the frying oil should be discarded or not. It is necessary to train food operators in fast food restaurants in order to apply proper methods of cooking and frying foods.

## 5 CONCLUSION

The present research results showed that the majority of fast food restaurants in Tehran use overdegraded frying oils, which are not discarded at the proper time. All the tasted discarded samples showed extremely high levels of degradation based on their *p*‐AV and TPC. There was no significant relationship between the oils’ rancidity parameters, replenishment, filtration, and frequency of filtration. Furthermore, there were positive correlations between TPC, viscosity, and red color. Considering the potential toxicity of the products of oxidation and hydrolysis of frying oils, any further utilization of used frying oils could be noticed as the public health hazard. It seems that, because of easy access and low price, the use of nonstandard oil, which is not suitable for frying in Iranian fast food restaurants, has increased. Prevention of oil exposure to extra heat, use of antioxidants, filtration or absorbents, pressure frying, avoidance of intermittent frying, and regular exchange of heated oil with fresh one are strategies that can be used by restaurant keepers to improve the condition. It is necessary to train food operators in fast food restaurants in order to apply proper methods of cooking and frying foods. Also, it would be better that policymakers design education programs concomitant with more vigilance from administrative organizations that may additionally help in the improvement.

## CONFLICT OF INTEREST

The authors declare that they have no conflicts of interest to disclose. This study received no specific grant from any funding sources (commercial or nonprofit sectors).

## ETHICAL CONSIDERATION

Ethical issues (including plagiarism, informed consent, misconduct, data fabrication and/or falsification, double publication and/or submission, and redundancy) have been completely observed by the authors.

## LIMITATION

The owners of fast food restaurants were hardly willing to collaborate.

## References

[fsn31072-bib-0001] (INSO), I. N. S. O. (1998). Animal and vegetable fats and oils – Determination of Lovibond colour, Vol. 5110.

[fsn31072-bib-0002] (INSO), I. N. S. O. (2016a). Animal and vegetable fats and oils‐Gas chromatography of fatty acid methyl esters – Part 2: Preparation of fatty acid methyl esters, Vol. 13126‐2.

[fsn31072-bib-0003] (INSO), I. N. S. O. (2016b). Edible fats & oils‐frying oil – Specifications and test methods, Vol. 4152.

[fsn31072-bib-0004] (INSO), I. N. S. O. (2018). Animal and vegetable fats and oils—Determination of peroxide value—Iodometric (visual) endpoint determination, Vol. 4179.

[fsn31072-bib-0005] Aalipour Hafshejani, F. , Mahdavi, F. , & Aalipour Hafshejani, E. (2015). Determination of peroxide value and visual color of Zoolbia and Bamieh oils in the holy Ramadan in Shahrekord. Journal of Shahrekord Uuniversity of Medical Sciences, 17(5), 74–82.

[fsn31072-bib-0006] Abedi, A.‐S. , Hosseini, H. , Mohammadi, A. , Abdollahi, Z. , Hajifaraji, M. , & Mousavi Khaneghah, A. (2016). Fatty acid (FA) compositions and trans content of frequently consumed edible oils and fats from iran market. Current Nutrition & Food Science, 12(1), 56–64.

[fsn31072-bib-0007] Aladedunye, F. A. (2015). Curbing thermo‐oxidative degradation of frying oils: Current knowledge and challenges. European Journal of Lipid Science and Technology, 117(11), 1867–1881. 10.1002/ejlt.201500047

[fsn31072-bib-0008] Al‐Harbi, M. , & Al‐Kahtani, H. A. (1993). Chemical and biological evaluation of discarded frying palm oil from commercial restaurants. Food Chemistry, 48(4), 395–401. 10.1016/0308-8146(93)90324-9

[fsn31072-bib-0009] Amirsardari, I. , Asadollahi, S. , & Eshaghi, M. R. (2018). Investigating on physicochemical properties and oxidative stability of palm free frying oil in comparison with palm containing frying oil. Journal of Food Science and Technology, 15, 245–255.

[fsn31072-bib-0010] AOCS.Ca5a‐40 . Official methods and recommended practices of the American Oil Chemists’ Society (AOCS). Champaign, IL: AOCS press.

[fsn31072-bib-0011] AOCS.Cd18‐90 . Official methods and recommended practices of the American Oil Chemists’ Society (AOCS). Champaign, IL: AOCS press.

[fsn31072-bib-0012] AOCS.Cd8b‐90 (2011). Official methods and recommended practices of the American Oil Chemists’ Society (AOCS), Vol. Cd 8b–90 Champaign, IL: AOCS Press.

[fsn31072-bib-0013] Arbabi, M. , & Deris, F. (2011). Determination of hydrogen peroxide index in the consumption of edible oils in fast food shops. Journal of Shahrekord Uuniversity of Medical Sciences, 13(3), 90–99.

[fsn31072-bib-0014] Bahadoran, Z. , Mirmiran, P. , Golzarand, M. , Hosseini‐Esfahani, F. , & Azizi, F. (2012). Fast food consumption in Iranian adults; dietary intake and cardiovascular risk factors: Tehran Lipid and Glucose Study. Archives of Iranian Medicine (AIM), 15(6), 346–351. 22642243

[fsn31072-bib-0015] Bailey, A. E. , & Shahidi, F. (2005). Bailey's industrial oil & fats products. New York, NY: John Wiley & Sons.

[fsn31072-bib-0016] Bansal, G. , Zhou, W. , Barlow, P. J. , Joshi, P. S. , Lo, H. L. , & Chung, Y. K. (2010). Review of rapid tests available for measuring the quality changes in frying oils and comparison with standard methods. Critical Reviews in Food Science and Nutrition, 50(6), 503–514. 10.1080/10408390802544611 20544441

[fsn31072-bib-0017] Boons, F. , & Mendoza, A. (2010). Constructing sustainable palm oil: How actors define sustainability. Journal of Cleaner Production, 18(16–17), 1686–1695. 10.1016/j.jclepro.2010.07.003

[fsn31072-bib-0018] Chen, W.‐A. , Chiu, C. P. , Cheng, W.‐C. , Hsu, C.‐K. , & Kuo, M.‐I. (2013). Total polar compounds and acid values of repeatedly used frying oils measured by standard and rapid methods. J Food Drug Anal, 21(1), 58–65.

[fsn31072-bib-0019] Crosa, M. J. , Skerl, V. , Cadenazzi, M. , Olazábal, L. , Silva, R. , Suburú, G. , & Torres, M. (2014). Changes produced in oils during vacuum and traditional frying of potato chips. Food Chemistry, 146, 603–607. 10.1016/j.foodchem.2013.08.132 24176387

[fsn31072-bib-0020] De Abreu, D. P. , Losada, P. P. , Maroto, J. , & Cruz, J. (2010). Evaluation of the effectiveness of a new active packaging film containing natural antioxidants (from barley husks) that retard lipid damage in frozen Atlantic salmon (*Salmo salar* L.). Food Research International, 43(5), 1277–1282. 10.1016/j.foodres.2010.03.019

[fsn31072-bib-0021] Farhoosh, R. , & Moosavi, S. (2010). Evaluating the performance of peroxide and conjugated diene values in monitoring the quality of used frying oils. Journal of Agricultural Science and Technology, 11, 173–179.

[fsn31072-bib-0022] Farrokhzadeh, H. , Ghorbani, E. , Hashemi, H. , Mohebat, L. , Hassanzadeh, A. , Yahay, M. , … Jaberi, H. (2013). Measurement of used oil rancidity indexes in the confectioneries and food shops. International Journal of Environmental Health Engineering, 2(1), 2302–4.

[fsn31072-bib-0023] FSSAI (2017). Reusing stale oil in fast food restaurants. India.

[fsn31072-bib-0024] Gertz, C. , & Stier, R. (2011). *Recommendations for used frying oils and fats* Paper presented at the 6th International Symposium on Deep‐Frying—Errors and Myths of Industrial and Catering Frying: German Society for Fat Research (DGF). Retrieved from http://www.dgfett.de/material/recomm.php.

[fsn31072-bib-0025] Gunstone, F. D. (2008). Oils and fats in the food industry. Oxford, UK: Wiley-Blackwell.

[fsn31072-bib-0026] Hassanzadazar, H. , Ghayurdoost, F. , Aminzare, M. , Mottaghianpour, E. , & Taami, B. (2018). Monitoring of Edible Oils Quality in Restaurants and Fast Food Centers Using Peroxide and Acid Values. Journal of Chemical Health Risks, 8(3), 209–216.

[fsn31072-bib-0027] International Organization for Standardization (ISO) (2011). Animal and vegetable fats and oils – Gas chromatography of fatty acid methyl esters. Vol. ISO 12966.

[fsn31072-bib-0028] Jamali, M. K. , Kazi, T. G. , Arain, M. B. , Afridi, H. I. , Jalbani, N. , Sarfraz, R. A. , & Baig, J. A. (2008). A multivariate study: Variation in uptake of trace and toxic elements by various varieties of *Sorghum bicolor* L. Journal of Hazardous Materials, 158(2–3), 644–651. 10.1016/j.jhazmat.2008.02.007 18353548

[fsn31072-bib-0029] Kang, Y. J. , & Yang, S. (2013). Integrated microfluidic viscometer equipped with fluid temperature controller for measurement of viscosity in complex fluids. Microfluidics and Nanofluidics, 14(3–4), 657–668. 10.1007/s10404-012-1085-5

[fsn31072-bib-0030] Karimi, S. , Wawire, M. , & Mathooko, F. M. (2017). Impact of frying practices and frying conditions on the quality and safety of frying oils used by street vendors and restaurants in Nairobi, Kenya. Journal of Food Composition and Analysis, 62, 239–244. 10.1016/j.jfca.2017.07.004

[fsn31072-bib-0031] Kubow, S. (1992). Routes of formation and toxic consequences of lipid oxidation products in foods. Free Radical Biology and Medicine, 12(1), 63–81. 10.1016/0891-5849(92)90059-P 1537572

[fsn31072-bib-0032] Kumar, D. , Singh, A. , & Tarsikka, P. S. (2013). Interrelationship between viscosity and electrical properties for edible oils. Journal of Food Science and Technology, 50(3), 549–554. 10.1007/s13197-011-0346-8 24425951 PMC3602556

[fsn31072-bib-0033] Li, Y. , Ngadi, M. , & Oluka, S. (2008). Quality changes in mixtures of hydrogenated and non‐hydrogenated oils during frying. Journal of the Science of Food and Agriculture, 88(9), 1518–1523. 10.1002/jsfa.3239

[fsn31072-bib-0034] Liu, M. , Xie, S. , Ge, J. I. , Xu, Z. , Wu, Z. , Ru, C. , … Sun, Y. U. (2016). Microfluidic assessment of frying oil degradation. Scientific Reports, 6, 27970 10.1038/srep27970 27312884 PMC4911549

[fsn31072-bib-0035] Masek, A. , Latos, M. , Chrzescijanska, E. , & Zaborski, M. (2017). Antioxidant properties of rose extract (*Rosa villosa* L.) measured using electrochemical and UV/Vis spectrophotometric methods. International Journal of Electrochemical Science, 12(11), 10994–11005.

[fsn31072-bib-0036] McSavage, J. T. , & Trevisan, S. (2001). The use and abuse of frying oil. Food Service Technology, 1(2), 85–92. 10.1046/j.1471-5740.2001.00013.x

[fsn31072-bib-0037] Melton, S. , Jafar, S. , Sykes, D. , & Trigiano, M. (1994). Review of stability measurements for frying oils and fried food flavor. Journal of the American Oil Chemists' Society, 71(12), 1301–1308. 10.1007/BF02541345

[fsn31072-bib-0038] Mohammadi, M. , Hajeb, P. , Seyyedian, R. , Hossein Mohebbi, G. , & Barmak, A. (2013). Evaluation of oxidative quality parameters in imported edible oils in Iran. British Food Journal, 115(6), 789–795. 10.1108/BFJ-Feb-2011-0035

[fsn31072-bib-0039] Moreira, R. , Castell‐Perez, M. , & Barrufet, M. (1999). Frying oil characteristics In Deep frying fundamentals and applications (pp. 33–74). Gaithersburg, MD: Aspen Publishers.

[fsn31072-bib-0040] Mozaffarian, D. , Katan, M. B. , Ascherio, A. , Stampfer, M. J. , & Willett, W. C. (2006). Trans fatty acids and cardiovascular disease. New England Journal of Medicine, 354(15), 1601–1613. 10.1056/NEJMra054035 16611951

[fsn31072-bib-0041] Navab Daneshmand, F. , & Ghavami, M. (2012). The effect of temperature and time on the production and decomposition of hydroperoxides in canola and soybean Oils. Journal of Food Technology and Nutrition, 9(1), 61–72.

[fsn31072-bib-0042] Oomen, C. M. , Ocké, M. C. , Feskens, E. J. , van Erp‐Baart, M.‐A.‐J. , Kok, F. J. , & Kromhout, D. (2001). Association between trans fatty acid intake and 10‐year risk of coronary heart disease in the Zutphen Elderly Study: A prospective population‐based study. The Lancet, 357(9258), 746–751. 10.1016/s0140-6736(00)04166-0 11253967

[fsn31072-bib-0043] Orthoefer, F. T. , & List, G. R. (2007). 12 - Dynamics of frying In Erickson M. D. (Ed.), Deep frying, chemistry, nutrition, and practical applications, (2nd ed, pp. 250‐275). Urbana, IL: AOCS Press.

[fsn31072-bib-0044] Oyagbemi, A. A. , Azeez, O. , & Saba, A. (2009). Interactions between reactive oxygen species and cancer: The roles of natural dietary antioxidants and their molecular mechanisms of action. Asian Pacific Journal of Cancer Prevention, 10(4), 535–544. 19827865

[fsn31072-bib-0045] Pizzino, G. , Irrera, N. , Cucinotta, M. , Pallio, G. , Mannino, F. , Arcoraci, V. , Bitto, A. (2017). Oxidative stress: Harms and benefits for human health. Oxidative Medicine and Cellular Longevity, 2017, 2302–13. 10.1155/2017/8416763 PMC5551541 28819546

[fsn31072-bib-0046] Pourmahmoudi, A. , Akbartabar Turi, M. , Poursamad, A. , Sadat, A. , & Karimi, A. (2008). Determination of peroxide value of edible oils used in restaurants and sandwich shops in Yasuj in 2006. Armaghane Danesh, 13(1), 115–123.

[fsn31072-bib-0047] Saghafi, Z. , Naeli, M. H. , Tabibiazar, M. , & Zargaraan, A. (2018). Zero‐trans cake shortening: Formulation and characterization of physicochemical, rheological, and textural properties. Journal of the American Oil Chemists' Society, 95(2), 171–183.

[fsn31072-bib-0048] Saguy, I. S. , & Dana, D. (2003). Integrated approach to deep fat frying: Engineering, nutrition, health and consumer aspects. Journal of Food Engineering, 56(2–3), 143–152. 10.1016/S0260-8774(02)00243-1

[fsn31072-bib-0049] Sebastian, A. , Ghazani, S. M. , & Marangoni, A. G. (2014). Quality and safety of frying oils used in restaurants. Food Research International, 64, 420–423. 10.1016/j.foodres.2014.07.033 30011669

[fsn31072-bib-0050] Shahidi, F. (2005). Bailey's industrial oil & fat products, Volume 5, Edible oil & fat products, processing technologies, 6th ed Hoboken, NJ: John Wiley & Sons, Inc.

[fsn31072-bib-0051] Tajali‐pour, M. , & Alikhani, M. (2012). Economic perspective of Tehran Province. Ministry of Economic and Financial Affairs

[fsn31072-bib-0052] Statistical Center of Iran , Household Expenditure and Income Statistics (2016). Available from: www.amar.org.ir [last accessed 11 May 2018].

[fsn31072-bib-0053] Uriarte, P. , & Guillén, M. (2010). Formation of toxic alkylbenzenes in edible oils submitted to frying temperature: Influence of oil composition in main components and heating time. Food Research International, 43(8), 2161–2170. 10.1016/j.foodres.2010.07.022

[fsn31072-bib-0054] van der Merwe, G. H. , du Plessis, L. M. , & Taylor, J. R. (2004). Changes in chemical quality indices during long‐term storage of palm‐olein oil under heated storage and transport‐type conditions. Journal of the Science of Food and Agriculture, 84(1), 52–58. 10.1002/jsfa.1609

[fsn31072-bib-0055] Vitrac, O. , Trystram, G. , & Raoult‐Wack, A. L. (2000). Deep‐fat frying of food: Heat and mass transfer, transformations and reactions inside the frying material. European Journal of Lipid Science and Technology, 102(8–9), 529–538. 10.1002/1438-9312(200009)102:8/9<529:AID-EJLT529>3.0.CO;2-F

[fsn31072-bib-0056] Zargaraan, A. , Mohammadi‐Nasrabadi, F. , Hosseini, H. , Salmani, Y. , Bahmaei, M. , & Esfarjani, F. (2018). Challenges of edible oils from farm to industry: Views of stakeholders. Food and Nutrition Bulletin, 1–12, 10.1177/0379572118813758 30518265

[fsn31072-bib-0057] Zhang, Q. , Saleh, A. S. , Chen, J. , & Shen, Q. (2012). Chemical alterations taken place during deep‐fat frying based on certain reaction products: A review. Chemistry and Physics of Lipids, 165(6), 662–681. 10.1016/j.chemphyslip.2012.07.002 22800882

